# A system based network approach to ethanol tolerance in *Saccharomyces cerevisiae*

**DOI:** 10.1186/s12918-014-0090-6

**Published:** 2014-08-08

**Authors:** Ceyda Kasavi, Serpil Eraslan, Kazim Yalcin Arga, Ebru Toksoy Oner, Betul Kirdar

**Affiliations:** 1 Department of Chemical Engineering, Boğaziçi University, Istanbul, Turkey; 2 Department of Bioengineering, Marmara University, Istanbul, Turkey

**Keywords:** Ethanol tolerance network, Candidate genes, Saccharomyces cerevisiae, Protein-protein interaction network

## Abstract

**Background:**

*Saccharomyces cerevisiae* has been widely used for bio-ethanol production and development of rational genetic engineering strategies leading both to the improvement of productivity and ethanol tolerance is very important for cost-effective bio-ethanol production. Studies on the identification of the genes that are up- or down-regulated in the presence of ethanol indicated that the genes may be involved to protect the cells against ethanol stress, but not necessarily required for ethanol tolerance.

**Results:**

In the present study, a novel network based approach was developed to identify candidate genes involved in ethanol tolerance. Protein-protein interaction (PPI) network associated with ethanol tolerance (tETN) was reconstructed by integrating PPI data with Gene Ontology (GO) terms. Modular analysis of the constructed networks revealed genes with no previously reported experimental evidence related to ethanol tolerance and resulted in the identification of 17 genes with previously unknown biological functions. We have randomly selected four of these genes and deletion strains of two genes (*YDR307W* and *YHL042W*) were found to exhibit improved tolerance to ethanol when compared to wild type strain.

The genome-wide transcriptomic response of yeast cells to the deletions of *YDR307W* and *YHL042W* in the absence of ethanol revealed that the deletion of *YDR307W* and *YHL042W* genes resulted in the transcriptional re-programming of the metabolism resulting from a mis-perception of the nutritional environment. Yeast cells perceived an excess amount of glucose and a deficiency of methionine or sulfur in the absence of *YDR307W* and *YHL042W,* respectively, possibly resulting from a defect in the nutritional sensing and signaling or transport mechanisms. Mutations leading to an increase in ribosome biogenesis were found to be important for the improvement of ethanol tolerance. Modulations of chronological life span were also identified to contribute to ethanol tolerance in yeast.

**Conclusions:**

The system based network approach developed allows the identification of novel gene targets for improved ethanol tolerance and supports the highly complex nature of ethanol tolerance in yeast.

## Background

*S. cerevisiae* can produce high concentrations of ethanol [1]–[3]. Therefore, it is commonly used for alcohol related brewing and fermentation technologies such as the production of alcoholic beverages, ethanol, and other products in food and chemical industries [2],[4].

During industrial bio-ethanol production processes, the increase in ethanol level acts as an inhibitor of microorganism growth and viability [5]–[8]. Therefore, yeast cells that have high growth ability under high ethanol concentrations are preferred in ethanol production processes [9]. Development of rational genetic engineering strategies leading both to the improvement of productivity and ethanol tolerance in yeast is considered to be very important for cost-effective bio-ethanol production [3],[10].

Several studies were carried out to understand the molecular basis of ethanol stress and ethanol tolerance in *S. cerevisiae*[2],[3],[9],[11]–[15]. The genes involved in ethanol sensitivity or resistance to ethanol and their functional categories could be identified by screening single gene knock out collection of yeast cells in the presence of ethanol [11],[13]–[15]. However, the reason for the improved ethanol resistance in the deletion strains remained unclear [3]. Investigations on the genome-wide transcriptional response of yeast cells to ethanol revealed a series of affected biological processes in response to ethanol stress. The genes involved in glycolysis and mitochondrial function were observed to be up-regulated and the genes involved in energy-demanding growth related processes were commonly found down-regulated under ethanol stress in several studies [2]. The genes associated with ethanol tolerance in yeast were also found to be linked to a broad range of different functional categories and biological functions including mitochondrial function, protein sorting, aromatic amino acid metabolism, vacuolar, vesicular and peroxisomal transport [3]. However despite the great efforts, the mechanisms underlying ethanol tolerance and ethanol toxicity are still not well known.

Ethanol tolerance and ethanol stress are considered to be two closely related characteristics of the overall effects of ethanol on the performance of yeast. Although there is no clear definition for these two different aspects of the same process, ethanol tolerance was defined as the strength or survival performance of yeast cells during the chronic ethanol exposure [2]. Studies on the identification of the genes that are up-regulated in the presence of ethanol indicated that these genes may be involved to protect the cells against ethanol stress, but not necessarily required for ethanol tolerance [11],[12]. It has been reported that the up-regulated genes in the presence of ethanol are commonly selected as targets for the construction of ethanol tolerant strains. However, the genes which do not display any change in their expression levels were found to be more important for the growth of yeast cell in the presence of ethanol [11]. Engineering of ethanol resistance by the modification of the binding properties of key transcriptional factors indicated that ethanol tolerance is associated with complex network of interactions [16]. Association of ethanol tolerance with an interplay of complex networks at the genome level also indicated that a system based approach would be required to elucidate and understand the tolerance and stress response mechanisms in yeast [3],[17].

Protein-protein interactions (PPIs) are essential for all biological processes and are deposited in publicly available databases. The PPI network analysis elicits rich system level information to understand the changes in cellular functions. Although functional relatedness can be achieved at any level of interaction; including physical interaction as well as co-expression, co-regulation and phenotypic behaviour, functional linkage networks are considered to be important to explore the general organization principles. Functionally related proteins are known to act usually in the form of modules of highly interacting proteins. The complex functions of the whole system can be investigated by these interactions within the modules which are considered as building blocks of biological systems. Human protein interactions have been used to identify potential disease causing proteins and targets for therapeutic interventions [18]–[21]. Systems based modular approaches were also extensively used for gene annotation, protein function prediction, identification of regulators and novel proteins in molecular pathways in yeast [22]–[27].

Several algorithms, based on edge-betweenness centrality [28], on shortest path distances within a network, on the Statistical-Algorithmic Method for Bicluster Analysis (SAMBA) [29] or on the approximation mapping of network nodes into Euclidean space followed by fuzzy c-means clustering [30] were developed to identify dense sub-graphs and functional modules within the PPI networks. The MCODE algorithm based on vertex weighting by local neighbourhood density [31] has been applied to various networks to detect densely connected regions, such as the functional modules associated with cell growth and cell cycle in *S. cerevisiae*[32].

The aim of the present study was to develop a novel network based modular approach to identify the genes which may have potential roles in ethanol tolerance in *S. cerevisiae* with the ultimate goal of finding novel targets for the rational design of ethanol tolerant strains. In order to identify candidate proteins involved in ethanol tolerance, a PPI network related to ethanol tolerance was reconstructed by integrating PPI data with Gene Ontology (GO) terms. Modular analysis of the constructed networks revealed genes with no previously reported experimental evidence related to ethanol tolerance. The hypotheses were tested experimentally by randomly selecting four deletion strains and then two of these strains with deletions of previously unknown biological functions (*YDR307W* and *YHL042W)*, were found to exhibit improved tolerance to ethanol when compared to the wild type strain. Furthermore, in order to shed light into the underlying mechanism of ethanol tolerance and biological function of these genes, whole genome level transcriptional response to the deletions of *YDR307W* and *YHL042W* in *S. cerevisiae* was investigated.

## Methods

### Network reconstruction

**Figure 1:**
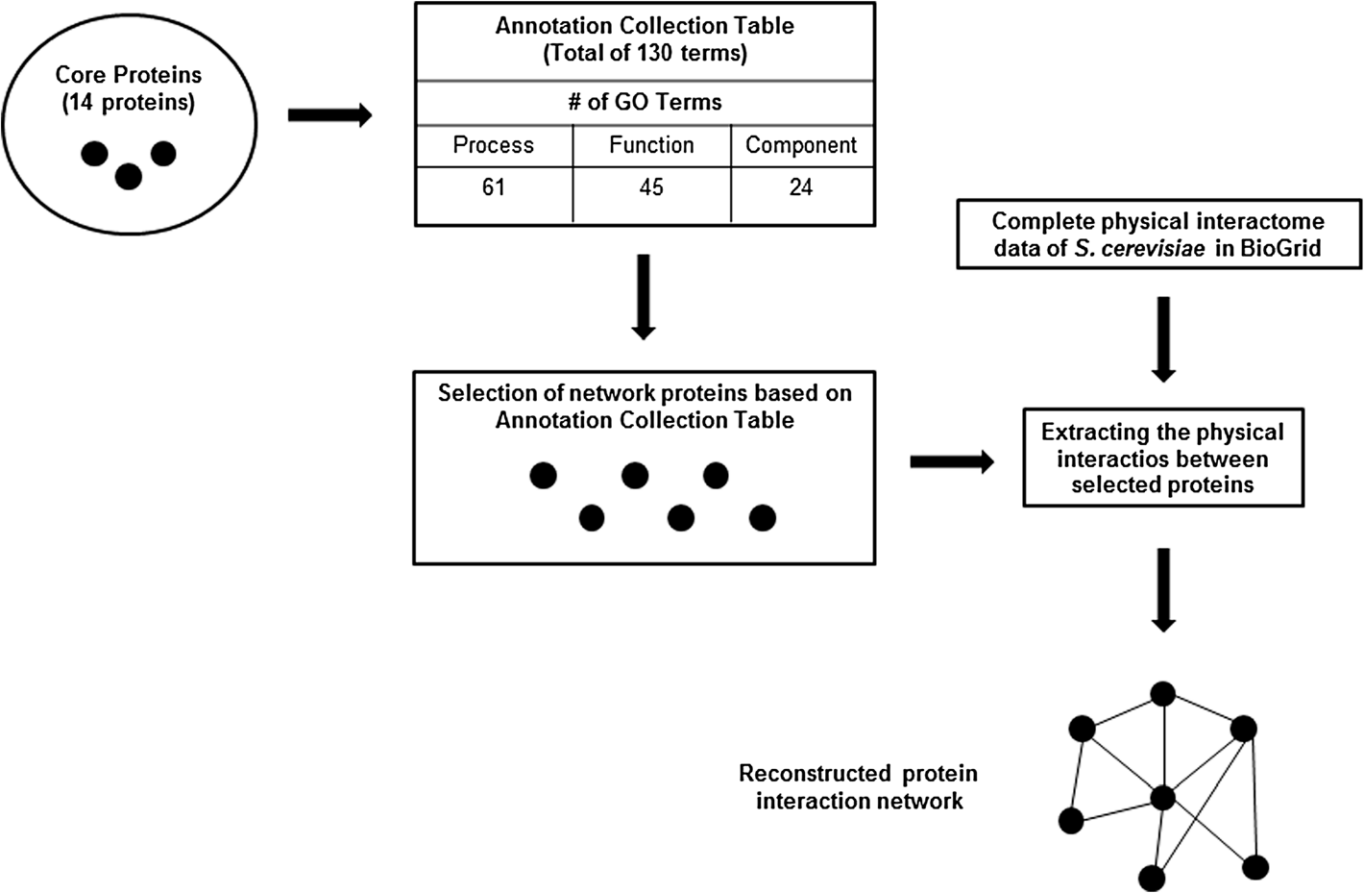
Schematic illustration of the network reconstruction algorithm, SPA.

Selective permissibility algorithm (SPA) that integrates protein-protein interaction data with the GO annotations was used to reconstruct a network constituted by the candidate proteins involved in ethanol tolerance as described by Arga *et al.*[33]. The reconstruction of Ethanol Tolerance Network (ETN) was initiated by selecting a total of 14 core proteins which were reported to be associated with ethanol tolerance from *Saccharomyces* Genome Database (SGD) (Table 1). Then an annotation collection table was created by pooling the GO annotations of core proteins in terms of cellular component, molecular function and biological process. This annotation collection table (Additional file 1) covered 130 GO annotations extracted out of a total of 4189 annotations (about 3%). In the reconstruction phase, a candidate protein was included to the network if all of three GO annotations (component/function/process) of the protein match to those in the annotation collection table. BioGrid [34] database release 3.1.73 was used to collect physical interactions between proteins. Figure 1 summarizes the reconstruction of ETN network. Finally, self-loops, duplicated edges, and significantly small connected components were eliminated.

**Table 1:** Core proteins of the network

**Core proteins**	**Description**	**Reference**
*URA7*	CTP synthase isozyme	[1]
*LAP3 (GAL6)*	Cysteine aminopeptidase	[1]
*EDE1*	Endocytic protein	[35]
*ELO1*	Elongase I (fatty acid elongation)	[35]
*TPS1*	Trehalose-6-phosphate synthase	[35]
*MSN2*	Transcriptional activator	[35],[36]
*DOG1*	2-deoxyglucose-6-phosphate phosphatase	[36]
*HAL1*	Cytoplasmic protein involved in halotolerance	[36]
*INO1*	Inositol-3-phosphate synthase	[36]
*OLE1*	Delta(9) fatty acid desaturase	[37]
*CYB5*	Cytochrome b5	[11]
*SFL1*	Repression of flocculation-related gene	[11]
*HSP26*	Heat shock protein	[38]
*RTC3*	Involved in RNA metabolism	[38]

### Network tuning

The reconstructed network was statistically tuned using the eigenvector centrality (EVC) metric. 100 random networks were generated by preserving the degree of each node. EVC values of ETN and random networks were computed in MATLAB 7.7 (MathWorks Inc.). For randomized networks, average values of EVC corresponding to each node were computed and a hypothesis testing was carried out for all nodes using two-tailed t-test with a confidence level of 99.99%. Consequently, the proteins in ETN, which are significantly different from those in random networks, were identified and the tuned ETN (tETN) was obtained by extracting physical interactions between these statistically significant nodes.

Topological properties of the networks, such as degrees, betweenness centralities, diameters, average shortest path lengths and clustering coefficients were examined by Network Analyzer [39] plug-in of Cytoscape [40].

### Module identification and functional enrichment

The highly connected protein subgroups of the reconstructed networks were identified via MCODE [31] plug-in of Cytoscape. In MCODE, loops were not included while scoring the networks and the degree threshold was set to 2. The node score threshold, K-core threshold, and maximum depth were set to 0.2, 2 and 100, respectively. The fluff parameter was turned off and the hair-cut parameter was turned on.

The GO enrichment analysis of modules, having at least 5 members, were performed via BINGO [41] plug-in (v2.44) of Cytoscape. The enrichment was evaluated by hypergeometric test using whole annotation as reference set; the multiple testing correction was made by Benjamini and Hochberg false discovery rate (FDR) correction; and the significance level was chosen to be 0.0001.

### Strains and media

The homozygous single gene deletion strains of *S. cerevisiae* from a genetic background of BY4743 (MATa/MATα his3Δ 0/his3Δ 0; leu2Δ /leu2Δ 0; met15Δ 0/MET15; LYS2/lys2Δ 0; ura3Δ 0/ura3Δ 0) used in this study (Table 2) were obtained from EUROSCARF [42] collection.

**Table 2:** Yeast strains used in this study

**Strain**	**Genotype**
ydr307wΔ/ydr307wΔ	BY4743; Mat a/α; his3Δ 1/his3Δ 1; leu2Δ 0/leu2Δ 0; lys2Δ 0/LYS2; MET15/met15Δ 0; ura3Δ 0/ura3Δ 0; YDR307w::kanMX4/YDR307w::kanMX4
yhl042wΔ/yhl042wΔ	BY4743; Mat a/α; his3Δ 1/his3Δ 1; leu2Δ 0/leu2Δ 0; lys2Δ 0/LYS2; MET15/met15Δ 0; ura3Δ 0/ura3Δ 0; YHL042w::kanMX4/YHL042w::kanMX4
ymr215wΔ/ymr215wΔ	BY4743; Mat a/α; his3Δ 1/his3Δ 1; leu2Δ 0/leu2Δ 0; lys2Δ 0/LYS2; MET15/ met15Δ 0; ura3Δ 0/ura3Δ 0; YMR215w::kanMX4/YMR215w::kanMX4
ypl264cΔ/ypl264cΔ	BY4743; Mat a/α; his3Δ 1/his3Δ 1; leu2Δ 0/leu2Δ 0; lys2Δ 0/LYS2; MET15/ met15Δ 0; ura3Δ 0/ura3Δ 0; YPL264c::kanMX4/YPL264c::kanMX4

The precultures were inoculated with a single colony of cells taken from yeast extract-peptone-glucose (YPD) plates and incubated in YPD medium (2% [w/v] D-glucose, 2% [w/v] peptone, 1% [w/v] yeast extract) at 30°C and 180 rpm.

### Determination of ethanol tolerance

The ethanol tolerance of *S. cerevisiae* strains was determined by means of colony-forming ability and viability. YPD medium was inoculated with a final OD of 0.05 at 600 nm and incubated at 30°C and 180 rpm for 24 h. In order to test the colony-forming abilities of the cells, samples taken from the liquid cultures were serially diluted and spotted onto YPD plates containing 5, 7, 10% (v/v) ethanol. The colony formation was monitored after 72 h incubation at 30°C. All experiments were carried out in duplicate.

Viability of cells was determined by colony-forming unit (CFU) method [43]. *S. cerevisiae* strains were grown in YPD medium with working volumes of 200 ml in 1 L flasks and a preculture at a volume fraction of 1% was used to inoculate the culture. After 10 h of incubation at 30°C under 180 rpm shaking, cells were treated with ethanol to have 8% (v/v) final ethanol concentration. Samples from the liquid cultures were 10^3^-fold diluted and spread onto YPD plates (two biological and two technical replicates) just before ethanol treatment and 2, 4, 6, 8 h after ethanol treatment. Then, the colony formation was monitored after 48 h incubation at 30°C. All cultures were presumed to be 100% viable at time 0 (just before ethanol treatment) and all CFU measurements were normalized accordingly.

### Determination of chronological life span

*S. cerevisiae* strains were grown in YPD medium. Samples taken from the cultures were 10^4^-fold diluted and spread onto YPD plates (two biological and two technical replicates) for each strain and time point. Colony formation was monitored after 48 h incubation at 30°C. The number of CFUs at Day 0 (72 h after inoculation) was considered to be the initial survival baseline, and all cultures were presumed to be 100% viable at this time point and all CFU measurements were normalized accordingly.

### Determination of biomass, glucose and ethanol concentrations

Dry cell weights (DCW) of steady state cultures were determined gravimetrically. Cells were first recovered from 1 ml culture samples through centrifugation, then washed with distilled water three times and dried at 70°C until constant weight was achieved. Reported DCW values are averages of five biological and three technical replicates for each data point.

The optical densities (ODs) of samples collected during the exponential phase of growth were used for the determination of maximum specific growth rates (μ_max_). Extracellular glucose and ethanol concentrations were determined enzymatically using enzymatic analysis kits (Sigma) as described by the manufacturer.

The two-tailed Student’s t-test assuming two samples had equal variances was used to identify the observed significant differences from the wild type strain. A *p*-value threshold of 0.05 was selected to determine the significant differences between the fermentation properties of the mutants and that of the reference strain.

### RNA extraction

*S. cerevisiae* strains were grown in YPD medium with working volumes of 200 ml in 1 L flasks. Cultures were kept with continuous shaking at 30°C and 180 rpm. In order to analyze the transcriptional response of cells to *YDR307W* and *YHL042W* gene deletions, samples taken at the mid-exponential phase of growth at an OD range of 0.85-0.95 were used. Samples harvested for the transcriptome analysis were immediately frozen in liquid nitrogen and stored at −80°C until RNA isolation. These experiments were carried out in triplicate.

RNA extraction was carried out in a robotic workstation, QIAcube (Qiagen, USA) using the enzymatic lysis protocol as described by Qiagen RNeasy mini kit (Cat no: 74106). The quality and quantity of the isolated RNA were checked via spectrophotometric analysis using UV–vis spectrophotometer (NanoDrop ND-1000, Thermo Fisher Scientific Inc., USA). RNA samples were subjected to second quality check step before used in microarray analysis. RNA integrity number (RIN) values were checked using a microfluidics-based platform (Bioanalyzer 2100 Agilent Technologies, USA) using RNA6000 Nanokit (Agilent Technologies, USA) and samples with RIN values 7–10 were processed.

### Microarray analysis

First-strand cDNA was synthesized and then converted into a double-stranded DNA initially from 100 ng of total RNA using GeneChip® 3’ IVT Express Kit (Affymetrix Inc., USA). This double stranded cDNA was used as a template for *in vitro* transcription and synthesis of biotin-labelled aRNA. The final product was purified and quantified using the Nanodrop spectrophotometer before fragmentation. The purification and fragmentation steps were carried out using GeneChip reagents. Fragmented aRNA was evaluated using Agilent 2100 Bioanalyzer (Agilent Technologies, Germany). Affymetrix Yeast 2.0 arrays were prepared for hybridization using the reagents supplied in the GeneChip® Hybridization, Wash, and Stain Kit. A total of 5 μg of aRNA was loaded onto 169 format arrays and hybridized for 16 hours. The chips were then loaded into a fluidics station for washing and staining using Affymetrix Command Console® Software (AGCC) 3.0.1 Fluidics Control Module with Mini_euk2v3. Finally, the chips were loaded onto the Affymetrix **GeneChip Scanner 3000**. All applications were performed as described in the Affymetrix GeneChip®Expression Analysis Technical Manual. The microarray data from this study have been submitted to ArrayExpress at the European Bioinformatics Institute under accession number [E-MTAB-2415] in compliance with MIAME guidelines.

### Microarray data acquisition and analysis

For the analysis of transcriptomics data, CEL files were normalized via quantile normalization using RMA [44] as implemented in the *affy* package [45] of R/Bioconductor suite of tools [46]. Significantly expressed genes were identified from the normalized log-expression values using the multiple testing option of LIMMA [47]. The *p*-value threshold of 0.01 and a fold change cutoff of 1.5 were maintained to identify significantly expressed genes. Genes satisfying both *p*-value and fold change thresholds, were determined as significantly and differentially expressed genes. Statistically significant genes were used as inputs for gene set enrichment analysis based on GO annotations. All GO enrichment analysis was performed via GO Term Enrichment tool in the Amigo software [48] by using SGD filter, with a maximum *p*-value of 0.01.

### Identification of reporter transcription factors

The regulatory pathways that were affected in response to the deletions of *YDR307W* and *YHL042W* genes were identified by Reporter Features analysis [49],[50] implemented in BioMet Toolbox [51]. Reporter Feature algorithm was used for the integration of regulome and transcriptome. Transcription factors (TFs) in the consensus list described in the Yeastract database [52] were considered for the construction of a regulatory network in yeast. Yeast regulatory network was constructed by extracting the TF - protein interactions with direct evidence in Yeastract database [53]. The 0.05 *p*-value threshold was maintained to determine Reporter TFs.

## Results and discussion

### Network reconstruction and tuning

A set of 14 proteins were used as the seed and SPA was recruited in a non-iterative manner to reconstruct the ethanol tolerance network (ETN). Then, the network was expanded by using strict criteria based on GO annotation terminology as described in the Methods section. Two core proteins (*DOG1* and *HAL1*) were eliminated from the network due to the lack of physical interactions. The resulting network, consisting of 1962 nodes and 7585 edges, was composed of 14 disconnected sub-networks. The largest connected component, consisting of 1933 nodes and 7569 edges, was further investigated as the final network ETN (Additional file 2).

Accuracy of PPI data is often criticized, since interactome data obtained from high-throughput experiments are thought to have a large number of false positives, i.e. the interactions that are spurious and do not occur in the cell [33],[54]–[56]. Therefore, in this study, the reconstructed network was further tuned statistically using EVC, which is an indicator of the importance of a node within the topological arrangement in a graph [57],[58]. Statistical tests were carried out for all nodes in ETN as described in the Methods section. Briefly, a hypothesis testing was carried out to check whether the EVC value of the protein in ETN is significantly different from that in random networks. Following the hypothesis testing, 8% of the nodes in ETN were considered as statistically insignificant and eliminated from the network together with their interactions (7% of the interactions in ETN). 1783 proteins (among 1933 proteins) with differential EVC values were extracted and the resulting network (tETN) consisted of 7037 physical interactions between these proteins (Additional file 3).

The topological analysis of the resultant networks (ETN and tETN) indicated that they have scale-free degree distributions (Additional file 4) and small-world properties characteristic to biological networks. Their topological parameters, such as the diameter, characteristic path length and clustering coefficient were in consistence with other protein interaction networks published in literature. The topological properties of the BioGrid network, constructed using all possible protein-protein interactions in *S. cerevisiae* reported in BioGrid database, were found to be significantly different from ETN and tETN; the diameter and characteristic path length were 40% and 27% smaller, and the clustering coefficient was 48% higher than those of the ETN and tETN (Additional file 5). Since the average clustering coefficient measures the possible modularity of the network [59]–[61], the average clustering coefficient curves were also analyzed for ETN, tETN (Additional file 4) and the BioGrid network (Additional file 6). For all reconstructed networks, the average clustering coefficient versus degree followed a power law distribution with C(k) ≈ k^-w^. The analysis revealed that ETN and tETN were hierarchical networks having w = 0.80 which in turn was higher than that of the BioGrid network (w = 0.65).

Consequently, analysis of the topological properties of the reconstructed networks revealed that the network tuning prevented random inclusion of proteins into the network and, reduced the number of the proteins without changing the overall topology.

After the completion of the network and during the experimental stage of the present study, 9 genes associated with ethanol tolerance phenotype were included in SGD. Four of these genes (*MIG3*, *PDR18*, *SPT15*, and *UTH1*) were found to be part of the tETN. Two (*MAP2*, *PDE2*) of the five remaining proteins have very few reported physical interactions and are not expected to be included into the tETN in the present study.

A comparison of the genes which were identified to be associated with ethanol tolerance in the four independent deletion library screening studies [11],[13]–[15] and the genes included into tETN resulted in 29% overlap. It was reported that only two mutants carrying *VPS36* and *SMI1* (members of the tETN) deletions were found to be ethanol sensitive across all four studies. This low number of common genes within various studies is possibly due to the differences in the experimental condition and/or strains [2]. Moreover, the comparison of 446 genes which were identified by examining the growth of a deletion library collection in the presence of ethanol [11] with three other single gene knock out studies [13]–[15] resulted in 18% overlap [2].

Metabolic process GO terms such as transcription, regulation of transcription and gene expression, chromatin modification and organization, oxidation-reduction, protein transport, cellular nitrogen compound, establishment of localization, protein folding, response to oxidative stress, oxoacid, ketone, carboxylic acid, glutamine family amino acid, cell aging, replicative cell aging, vesicle mediated transport, response to stress, amino amino acid, cellular carbohydrate carbohydrate, alcohol, fatty acid, lipid, trehalose, sterol, steroid and ergosterol acetyl CoA metabolic processes were found to be significantly associated (*p*-value < 0.0001) with tETN. Since the majority of these biological process terms were already reported to be associated with ethanol tolerance [2], this analysis provided further support about the functional linkage between ethanol tolerance and tETN.

Aging and ethanol tolerance were not reported previously to be related. However, tETN constructed in this study, was significantly enriched with GO biological process terms including “aging”, “cell aging” and “replicative cell aging”. This finding indicated a possible association between ethanol tolerance and aging. A variety of cellular mechanisms, including metabolism, energy production, and stress responses, are involved in aging. The association between ethanol tolerance mechanism and other stresses, such as oxidative stress that causes oxidative DNA damage and DNA replication stress during aging also suggests a potential link between cell aging and ethanol tolerance.

The manual investigation of genes related to proline and tryptophan biosynthesis revealed the presence of 4 genes involved in proline biosynthesis (*PRO2*, *PRO3*, *PUT2*, and *YHR033W*) and 4 genes involved in tryptophan biosynthesis (*TRP1*, *TRP3*, *TRP4*, and *TRP5*) in tETN. An association between genes related to proline and tryptophan biosynthesis and ethanol tolerance was already reported [9]. The presence of most of the proline and tryptophan biosynthesis genes (8 out of 10) in tETN provided additional support that both processes are important for ethanol tolerance.

### Modular analysis and candidate genes for ethanol tolerance

Analysis of tETN by MCODE [31] plug-in of Cytoscape revealed the presence of 35 modules within tETN (Additional file 7). Clusters having at least 5 members (16 clusters) were further investigated and significantly enriched GO biological process terms were identified (Additional file 8). Four clusters (Clusters 1, 2, 6 and 9) were significantly enriched with transcription, regulation of transcription. Cluster 3 was found to be associated with RNA processing, RNA metabolic process and ribosome biogenesis, Cluster 4 with sterol, ergosterol, steroid, lipid metabolic processes, alcoholic metabolic process, and Golgi vesicle transport, Cluster 5 with chromatin modification, Cluster 7 with vitamin B6, thiamin and glutamine metabolic processes, Cluster 10 with transport, localization, pH reduction and regulation, protein folding, ion homeostasis and vacuolar acidification, Cluster 13 with trehalose processes, Cluster 18 with cell cycle regulation of transcription and Cluster 24 with coenzyme/cofactor metabolic processes, and glutathione and sulfur metabolic processes. Golgi vesicle transport and ER to Golgi transport GO process terms were identified to be significantly associated with Clusters 4, 5 and 10. Three clusters (Clusters 11, 12 and 23) were not associated with any specific GO process terms.

17 proteins with unknown biological functions were identified and four proteins (proteins encoded by *YDR307W*, *YHL042W* (members of Cluster 10) and *YMR215W*, and *YPL264C* (members of Cluster 4)) (Additional file 9) were randomly selected as the first targets to test experimentally. *YDR307W* (*PMT7*) is a putative mannosyltransferase similar to Pmt1p with a potential role in protein O-glycosylation [62]; *YHL042W* is a putative protein of unknown function, member of the DUP380 subfamily [63],[64]; *YMR215W* (*GAS3*) is a low abundance, possibly inactive member of the GAS family of GPI-containing proteins, a putative 1,3-beta-glucanosyltransferase with similarity to other GAS family members [65]–[68]; *YPL264C* is a putative membrane protein of unknown function [69],[70].

### Ethanol tolerances of *S. cerevisiae* strains

The ethanol tolerance of yeast cells carrying homozygous deletion of these four genes were first investigated in the presence of various concentrations of ethanol. Comparison of the colony-forming abilities of these strains with the wild type did not show any difference in the presence of 5% (v/v) ethanol. However, colony forming abilities of the ydr307wΔ/ydr307wΔ and yhl042wΔ/yhl042wΔ strains were found to be higher in media containing 7 and 10% (v/v) ethanol when compared with that of the wild type strain. Colony formation was observed with 10^6^ fold and 10^5^ fold diluted cultures of ydr307wΔ/ydr307wΔ and yhl042wΔ/yhl042wΔ strains, respectively. Colony formation could only be observed with 10^4^ fold diluted cultures of wild type, ypl264cΔ/ypl264cΔ and ymr215wΔ/ymr215wΔ strains in media containing 10% (v/v) ethanol (Additional file 10).

Comparison of the growth profiles of wild type strain in YPD supplemented with varying amounts of ethanol indicated that the maximum specific growth rates were 0.438 h^−1^ and 0.0778 h^−1^ in media containing 0% (v/v) and 8% (v/v) ethanol, respectively (Additional file 11). Since the growth was very poor and at undetectable levels in the presence of 10% (v/v) ethanol, 8% (v/v) ethanol was selected for further investigations.

**Figure 2:**
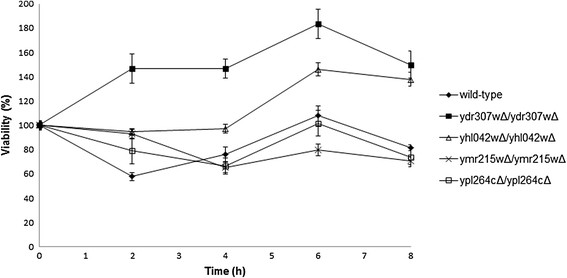
**Viability of*S. cerevisiae*strains after 8% (v/v) ethanol treatment.** The error bars denote the standard deviations of the two biological and two technical replicates.

Viability analysis of the four deleted strains after ethanol treatment indicated that all deletion mutants showed 1.2 - 2.5 fold higher viability than the wild type strain after 2 hours of ethanol treatment, a prolonged ethanol treatment of the ymr215wΔ/ymr215wΔ and ypl264cΔ/ypl264cΔ strains resulted in 0.7 and 0.9 fold less viability than the wild type strain, respectively. However, the ydr307wΔ/ydr307wΔ and yhl042wΔ/yhl042wΔ strains displayed 1.7 and 1.4 fold higher viabilities than the wild type strain after 6 hours of ethanol treatment, respectively (Figure 2). The viability profiles of the wild type, ydr307wΔ/ydr307wΔ and yhl042wΔ/yhl042wΔ strains were analyzed by ANOVA and highly significant differences in viability were observed between the strains. The fact that the deletions of *YDR307W* and *YHL042W* genes significantly increased (*p*-value < 0.005) the viability of these strains for prolonged treatment of ethanol indicated that these genes were indeed associated with ethanol tolerance in *S. cerevisiae* and were selected for further studies.

The growth profiles of the mutants were compared with those of the wild type strain in the presence of 8% (v/v) ethanol. The maximum specific growth rates of the ydr307wΔ/ydr307wΔ and yhl042wΔ/yhl042wΔ strains were 0.0859 h^−1^ and 0.0824 h^−1^, respectively. Although, no significant differences were observed in the growth rates, the mutants reached about 12–14% higher stationary biomass concentrations than that of the wild type strain (*p*-value < 0.001) (Additional file 12).

### Fermentation characteristics of *S. cerevisiae* strains

The fermentation characteristics of the wild type, ydr307wΔ/ydr307wΔ and yhl042wΔ/yhl042wΔ strains were investigated in the absence of ethanol (Table 3).

**Table 3:** **Comparison of fermentation parameters of** ***S. cerevisiae*** **strains**

**Parameter**	**Strains**		
	**Wild type**	**ydr307wΔ/ydr307wΔ**	**yhl042wΔ/yhl042wΔ**
Final DCW (g/L)	5.462	5.458	5.457
Max. ethanol conc. (g/L)*	6.59	6.15	7.14
Total glucose utilized (g/L)	18.46	18.36	18.46
μ_max_ (h^−1^)	0.453	0.454	0.451
Yps (g ethanol/g glucose)*	0.357	0.335	0.387
Ypx (g ethanol/g biomass)	1.207	1.127	1.308

(*) indicates significantly different (*p*-value < 0.005) fermentation parameters of mutants when compared to the wild type strain.

**Figure 3:**
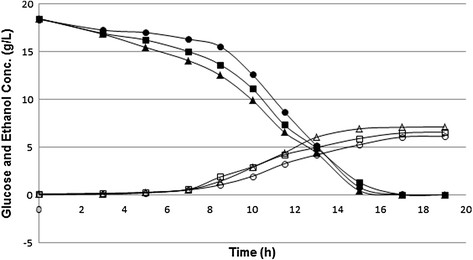
**Extracellular glucose and ethanol concentrations.** Consumption of glucose (filled symbols) and production of ethanol (open symbols) by the wild type (■,□), ydr307wΔ/ydr307wΔ (●,○) and yhl042wΔ/yhl042wΔ (▲,Δ) strains.

The maximum biomass concentrations as well as their μ_max_ values were found to be very similar for all strains. However, the maximum ethanol concentrations observed by the ydr307wΔ/ydr307wΔ and yhl042wΔ/yhl042wΔ strains were 7% lower (*p*-value = 0.003) and 8% higher (*p*-value = 0.002) than that of the wild type strain, respectively. The ethanol yield on biomass was highest in yhl042wΔ/yhl042wΔ strain. The glucose was totally consumed by all strains at end of 18 hours of fermentation. However, the remaining glucose was found to be 14% higher (*p*-value = 0.02) in ydr307wΔ/ydr307wΔ strain than that of the wild type strain at t = 8.5 h (Figure 3).

It has to be noted that the deletion of *YHL042W* leading to an improved ethanol tolerance has also resulted in an increase in the ethanol production. Although the efficiency of ethanol production in ethanol tolerant strains is important, most of the toxicity studies rely on the viability analysis of the strains in the presence of ethanol [1],[11],[35],[37],[38]. However, the engineered yeast strains with both improved ethanol tolerance and ethanol production were reported with 8% [71] and 15% [16] ethanol yields or reaching to 25.7% [72] and 6% [73] ethanol concentrations.

### Global transcriptional response to *YDR307W* and *YHL042W* gene deletions

In order to shed light into the molecular mechanisms involved in the improvement of ethanol tolerance as well as to assign a possible function to these two unknown genes, the genome-wide transcriptional response of yeast to the deletion of *YDR307W* and *YHL042W* was investigated and compared with their parent, *S. cerevisiae* BY4743.

**Figure 4:**
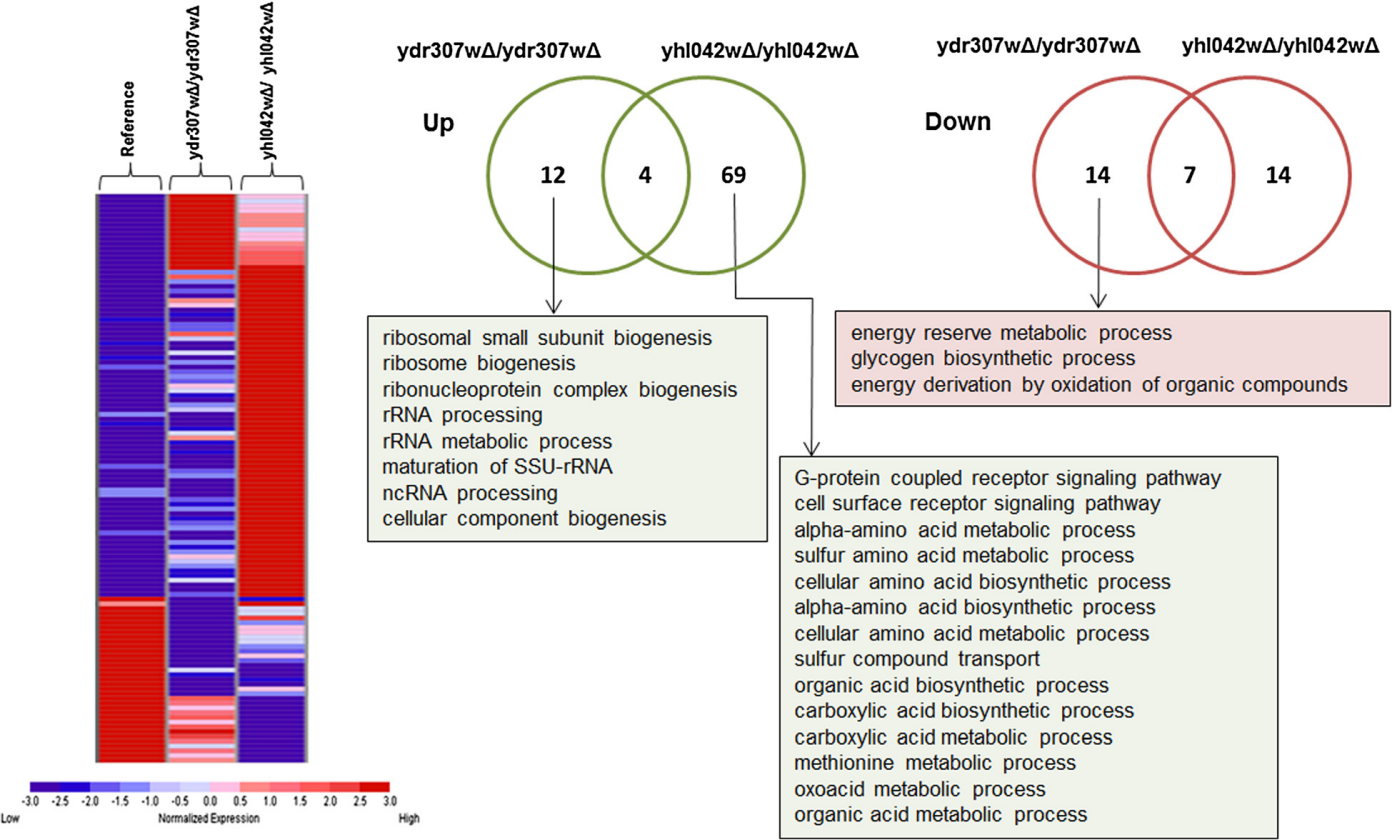
**Heat map representation and enriched GO biological process terms.** The heat map representation of the genes, which were significantly and differentially expressed in response to gene deletion; and the enriched GO biological process terms (*p*-value < 0.01) among the up- and down-regulated genes.

The samples for transcriptomic analysis were taken at the mid-exponential phase of growth from cultures grown in YPD. When compared with the reference strain, a total of 37 and 94 genes displayed significantly altered expression levels in response to *YDR307W* and *YHL042W* gene deletions, respectively and GO process terms which are significantly associated with these genes were identified (Figure 4). Furthermore, the integration of the gene regulatory network with the transcriptome data using Reporter Features algorithm [49],[50] revealed the key transcriptional factors around which significant transcriptional changes occur.

In response to *YDR307W* gene deletion, 12 genes were up-regulated and they were significantly enriched for growth associated processes including ribosome biogenesis (*p*-value < 0.00001) and rRNA processing (*p*-value < 0.001) (Figure 4). Ribosome biogenesis was reported to be a critical factor for yeast cells during industrial environmental stress [74] and was found to be carefully regulated in ethanol adapted and ethanol tolerant yeast cells under ethanol stress [75],[76].

14 genes were significantly repressed in a response to this deletion. The down-regulated genes were found to be significantly enriched with energy reserve metabolic process (*p*-value < 0.001). The genes involved in glycogen synthesis and accumulation, namely, *GSY1*, *GLC3*, *IGD1*, and *RGI1* encoding a protein of unknown function involved in energy metabolism under respiratory conditions were identified within this group. Manual inspection of the remaining down-regulated genes which are not statistically enriched with a specific GO biological process term, revealed that *MTH1* encoding a negative regulator of glucose sensing signal transduction pathway and, *TFS1* which encodes an anionic phospholipid binding protein involved in the regulation of the protein kinase A (PKA) signaling pathway as well as in the inhibition of vacuolar protease CPY were significantly repressed in response to this deletion. *CYC7* encoding an electron carrier involved in cellular respiration, *DCS2* involved in the regulation of stress, *SPI1* encoding a GPI-anchored cell wall protein involved in weak acid resistance and *STF2* involved in resistance to dessication stress were also identified among the down-regulated genes. The majority of these down-regulated genes, namely *STF2* and its paralog *TMA10*, *RTC3* encoding a protein of unknown function, *CYC7* and *TFS1*, were reported to be induced in response to DNA replication stress.

Reporter TF analysis indicated that significantly important transcriptional changes occur around the stress induced transcription factors Xbp1p and Sko1p, and transcription factors associated with filamentous growth (Mga1p and Flo8p) in response to the deletion of *YDR307W* in yeast.

Induction of the genes involved in ribosome biogenesis and repression of the genes involved in glycogen biosynthesis indicate a mis-perception of the glucose in the environment resulting possibly from a defect in the glucose sensing and signaling pathways or transport mechanism.

In response to the deletion of *YHL042W*, 69 genes were identified to be up-regulated in yeast. The genes *MFA1, MFA2, STE2*, *STE5*, *STE18*, *FUS3*, *FAR1*, and *RGS2* were significantly associated with G-protein coupled receptor signaling pathway (*p*-value < 0.0001). G-protein alpha-subunit binding process was already reported to be involved in ethanol tolerance [3]. *STE2* encodes the receptor for alpha-pheromone, *STE18* encodes the gamma subunit of the trimeric G protein, *STE5* encodes a MAPK scaffold, and *FUS3* which encodes a MAPK, phosphorylates the cell regulator Far1p. This protein is a pheromone activated Cdk-inhibitor (CKI) and was reported to be involved in the cell cycle arrest [77]. *RGS2*, which encodes a negative regulator of glucose induced cAMP signaling pathway, is reported to activate the GTPase activity of the alpha subunit of the trimeric G protein with a potential relevance to longevity [78].

**Figure 5:**
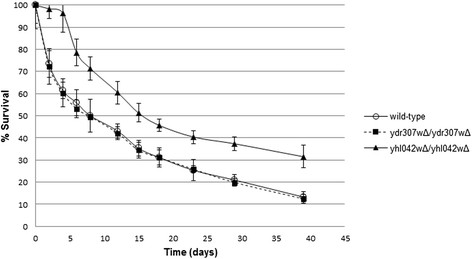
**Survival curves of*S. cerevisiae*strains.** The error bars denote the standard deviations of the two biological and two technical replicates.

The chronological life span of yhl042wΔ/yhl042wΔ strain was found to be longer than that of the wild type. The mean life span (the day on which survival reaches 50%) of yhl042wΔ/yhl042wΔ was calculated to be 87.5% higher than that of the wild type strain (Figure 5).

The genes which are induced in a response to this deletion were also significantly enriched for sulfur amino acid metabolic process (*p*-value < 0.001), methionine metabolic process (*p*-value < 0.01) and carboxylic acid metabolic process (*p*-value < 0.01). Methionine, cysteine and S-adenosyl methionine (SAM) are synthesized by S-assimilation or methionine pathway and have important roles in metabolism, redox reactions, initiation of protein synthesis and cell growth. Metabolites such as glutathione and acetyl Co-enzyme A contain sulfur atoms. The synthesis of phospholipids and polyamines are dependent on the transfer of methyl or aminopropyl groups from SAM. *MET32*, which is a transcriptional factor involved in the regulation of methionine biosynthetic genes, was identified among these up-regulated genes.

The genes (*STR3*, *MET6*, *SER2*, *MHT1*, *MET1*, *MET2*) encoding enzymes that catalyze at least one step in the biosynthesis of serine, homocysteine and methionine and *SAM1* encoding an enzyme for the conversion of methionine to S-adenosyl methionine were up-regulated. *HIS7* encoding the enzymes involved in the biosynthesis of histidine and in the conversion of L-glutamine to L-glutamate; *ARG1* which encodes arginosuccinate synthetase that catalyzes the formation of L-argininosuccinate from citrulline and L-aspartate in the arginine biosynthesis pathway; and *DUR1,2* which is involved in the allantoin degradation pathway that converts allantoin to ammonia and carbon dioxide were also found among the up-regulated genes. *ARG1* and *DUR1,2* play roles in the utilization of citrulline and allantoin as nitrogen sources, respectively. Additionally, *SRY1* encoding 3-hydroxyaspartate dehydratase which catalyzes the deamination of L-threo-3-hydroxyaspartate to form oxaloacetate and ammonia; *CIT2* encoding citrate synthetase which catalyze the condensation of acetyl co-enzyme A to oxaloacetate to form citrate; *MTD1* encoding 5,10-methylenetetrahydrafolate dehydrogenase involved in the biosynthesis of folate; and *FAA2* encoding medium chain fatty acyl-CoA synthetase involved in the activation of imported fatty acids were induced in response to this deletion.

The integration of the regulatory network with the transcriptomic response of the *YHL042W* deleted strain revealed 13 transcription factors (p < 0.05) around which the most significant transcriptional changes occur. Met32p, Met31p, Met28p, and Cbf1p involved in the regulation of sulfur metabolism and/or the regulation of methionine biosynthetic genes; Dal81p, a positive regulator of genes in multiple nitrogen degradation pathways; Dal82p, a positive regulator of allophanate inducible genes; Bas1p and Stp2p regulating amino acid metabolism; Yap1p required for oxidative stress tolerance; Zap1p involved in the transcriptional regulation of certain genes in the presence of zinc; Mot3p involved in repression of hypoxic genes during aerobic growth and ergosterol biosynthetic genes in response to hyperosmotic stress; Cst6p, a basic leucine zipper transcription factor; and Rim101p involved in alkaline responsive gene repression and cell wall assembly were the identified reporter transcription factors.

The deletion of *YHL042W* seems to mimic sulfur or methionine deprivation of auxotrophic yeast strains. Sulfur or methionine deprivation was reported to repress the iron import due to the lack of sulfur atoms for Fe-S biogenesis [79]. Manual investigation of the up-regulated genes which are not significantly enriched with a specific GO biological process term, revealed that *FIT2* which encodes a protein involved in the retention of siderophore-iron in the cell wall and *FRE2* which encodes a ferric/cupric reductase involved in the reduction of sidereophore bound iron and in the oxidation of copper prior to uptake were induced in response to this deletion. Additionally, *IZH4* encoding a membrane protein involved in zinc ion homeostasis with a possible role in sterol metabolism and *ZRT1* and *ZRT2* encoding high- and low-affinity zinc transporters were also found to be significantly over-expressed in this deletion strain. Furthermore, *MMP1* and *SAM3* which encode high-affinity S-methylmethionine and S-adenosylmethionine permeases, *MUP1* and *MUP3* which encode high- and low-affinity methionine permeases, *YNL024C* which encodes a putative methyl transferase and *SSU1* which encodes a plasma membrane sulfite pump required for efficient sulfite efflux were significantly and differentially up-regulated in response to this deletion. The genes encoding Jen1p, a membrane located monocarboxylate/proton symporter involved in the transport of high affinity carbon sources, Mch1p with a similarity to a mammalian monocarboxylate permease, Atr1p, a multidrug efflux pump and Opt1p which is a proton coupled oligopeptide transporter involved in the transport of glutathione were also observed to be significantly induced in this deleted strain.

All these findings provide supportive evidence that the deletion of *YHL042W* in yeast is experienced as the methionine or sulfur deprivation by these yeast cells although there was no missing environmental nutritional factor from the environment.

14 genes were found to be significantly and differentially down-regulated in response to the deletion of *YHL042W*. However, they are not found to be significantly enriched with any biological process term. Manual investigation of the down-regulated genes revealed that *ARO9* which encodes the first step of the catabolic metabolic process of aromatic amino acids and *ALD3* which encodes cytoplasmic aldehyde dehydrogenase were significantly repressed in response to this deletion. Furthermore, *GND1* encoding phosphogluconate dehydrogenase that is involved in the regeneration of NADPH; *SOL4* encoding 6-phosphogluconolactonase that functions in pentose-phosphate pathway; and *NQM1* which encodes a transaldolase of unknown function were also found to be significantly down-regulated in this deleted strain. Another member of DUP380 sub-family, *COS8*, which has a possible role in the unfolded protein response and *HSP26* involved in the suppression of unfolded protein aggregation were also identified among the down-regulated genes in a response to the deletion of *YHL042W.* Moreover, *TIS11* involved in iron homeostasis, *TSA2* encoding a stress inducible cytoplasmic thioredoxin peroxidase involved in the removal of oxygen, nitrogen and sulfur species using thioredoxin as hydrogen donor, and *YER053C-A* encoding a protein of unknown function were also identified among the down-regulated genes. The amount of the proteins encoded by *TIS11*, *TSA2* and *YER053C-A* was reported to be increased in response to DNA replication stress [80].

7 genes were commonly repressed and 4 genes commonly induced in both strains in response to the deletions of *YDR307W* and *YHL042W*. However, they are not found to be significantly enriched with any biological process term. Manual inspection of the induced genes (*TOD6*, *SYO1*, *DHR2*, and *DBP2*) indicated that all these genes are involved in ribosome biogenesis and RNA processing, and the majority of the commonly down regulated genes including *TMA10*, *SOL4*, *FMP16*, *HSP42* and *RTN2* were reported to be induced under DNA replication stress.

## Conclusions

There is an ever-growing interest in research towards understanding the metabolic processes and pathways associated with ethanol tolerance in *S. cerevisiae.* Within the framework of the present study, a network approach has been developed to identify the candidate genes leading to ethanol tolerance in *S. cerevisiae*. tETN harboring all candidate tolerance genes was found to be significantly enriched with biological process terms reported so far. The topological analysis indicated the hierarchical nature of the network and modular analysis identified 17 genes with unknown biological function. Investigations of the deletion strains of the randomly selected four of these genes, two strains carrying the deletions of *YDR307W* and *YHL042W* showed an improved ethanol tolerance. Although the deletion of other two genes (*YPL264C* and *YMR215W*), that were also identified as candidate, resulted in decreased viability after prolonged treatment of ethanol, the effect of the over-expression of both genes needs to be further investigated to understand the association of these genes with ethanol tolerance in yeast.

The genome-wide transcriptomic response of yeast cells to the deletions of *YDR307W* and *YHL042W* under normal conditions, in the absence of ethanol revealed that the deletion of *YDR307W* and *YHL042W* genes resulted in the transcriptional re-programming of the metabolism due to a mis-perception of the nutritional environment. Induction of the genes involved in ribosome biogenesis and repression of the genes involved in glycogen biosynthesis possibly resulting from a defect in the glucose sensing and signaling pathways or transport mechanism in the absence of *YDR307W* requires further investigations to shed light into the molecular function of this gene in yeast. Yeast cells carrying homozygous deletions of *YHL042W* seem to have a deficiency in the sensing and signaling or transportation of methionine or sulfur mechanism. Since the ribosome biogenesis decreases in the presence of ethanol, up-regulation of ribosome biogenesis might be an important factor contributing to the improvement of ethanol tolerance in both cases. In the case of the yhl042wΔ/yhl042wΔ, the elongation of the chronological life span may be an important contributing factor in ethanol tolerance.

The present study supported further the highly complicated nature of ethanol tolerance in yeast. High-throughput technologies should be used to test the ethanol tolerance of deletion and over-expression strains of all candidate genes. Furthermore, integrative analysis of the ethanol tolerant strains at different omics levels in the absence and presence of ethanol may shed light into the mechanisms leading to the improvement of ethanol tolerance.

## Competing interests

The authors declare that they have no competing interests.

## Authors’ contributions

BK and ETO defined the research theme; BK and CK conceived and designed the methods and experiments, CK carried out the laboratory experiments and performed the data analysis, SE carried out the microarray experiments, KYA contributed by providing a methodological perspective. BK and CK wrote the manuscript and all authors have read and approved the final manuscript.

## Additional files

## Supplementary Material

Additional file 1: Table S1. Annotation collection table of the core proteins. Click here for file

Additional file 2: Table S2. Protein-protein interactions of ETN. Click here for file

Additional file 3: Table S3. Protein-protein interactions of tETN. Click here for file

Additional file 4: Figure S1. Connectivity and average clustering coefficient distributions of the reconstructed networks A) ETN B) tETN. Click here for file

Additional file 5: Table S4. Topological properties of the reconstructed networks. Click here for file

Additional file 6: Figure S2. Connectivity and average clustering coefficient distributions of BioGrid Network. Click here for file

Additional file 7: Table S5. MCODE results for tETN. Click here for file

Additional file 8: Table S6. Significantly enriched GO biological process terms of tETN clusters having at least 5 members. Click here for file

Additional file 9: Figure S3. The clusters of four proteins (*YDR307W*, *YHL042W*, *YMR215W*, and *YPL264C*) that were selected as the first targets to test experimentally A) Cluster 4 B) Cluster 10. Click here for file

Additional file 10: Figure S4. Colony-forming ability of *S. cerevisiae* cultures. Click here for file

Additional file 11: Figure S5. Growth of the wild type strain in YPD supplemented with different concentrations of ethanol. Click here for file

Additional file 12: Figure S6. Growth of *S. cerevisiae* strains in YPD supplemented with 8% (v/v) ethanol. Click here for file
