# Supplementary figures and images for: A system based network approach to ethanol tolerance in Saccharomyces cerevisiae
Source: BMC Syst Biol. 2014 Aug 8;8:90. doi: 10.1186/s12918-014-0090-6 (PMC4236716; doi:10.1186/s12918-014-0090-6)

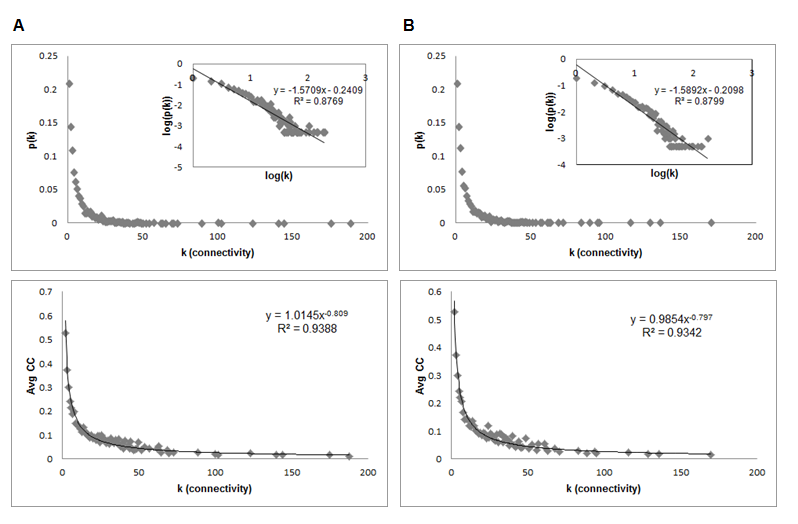

Supplement: Additional file 4: Figure S1. — Connectivity and average clustering coefficient distributions of the reconstructed networks A) ETN B) tETN. [file s12918-014-0090-6-S4.tiff]

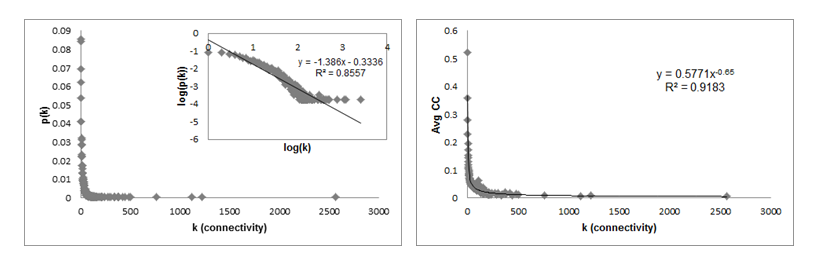

Supplement: Additional file 6: Figure S2. — Connectivity and average clustering coefficient distributions of BioGrid Network. [file s12918-014-0090-6-S6.tiff]

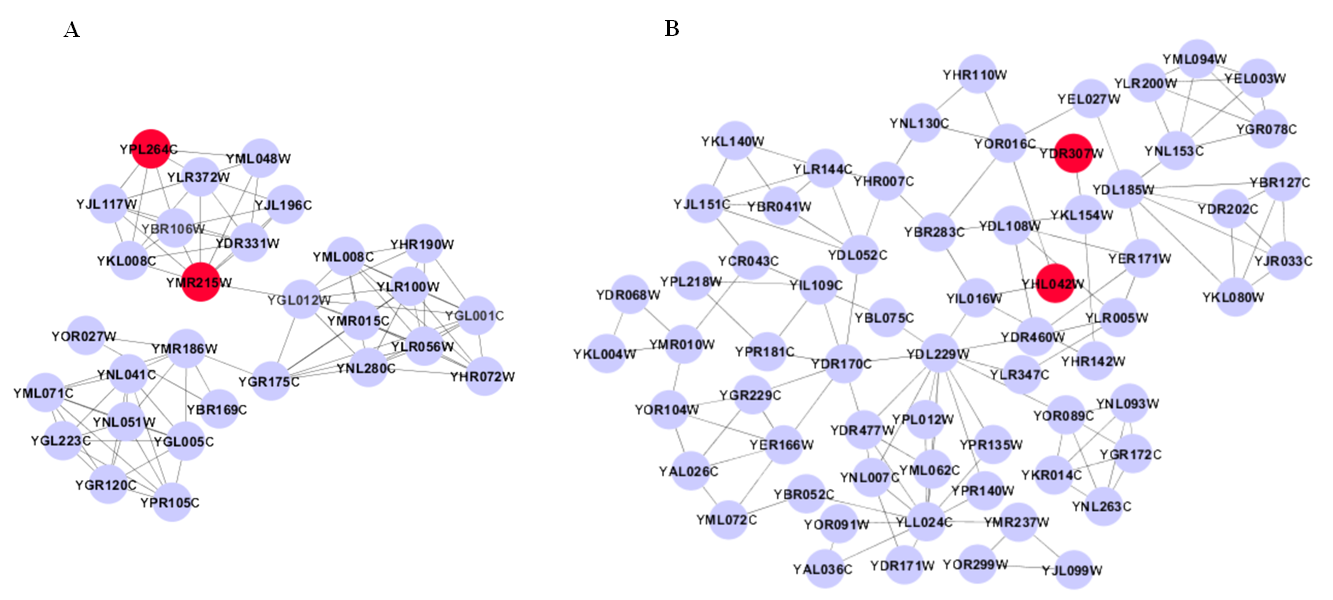

Supplement: Additional file 9: Figure S3. — The clusters of four proteins (YDR307W, YHL042W, YMR215W, and YPL264C) that were selected as the first targets to test experimentally A) Cluster 4 B) Cluster 10. [file s12918-014-0090-6-S9.tiff]

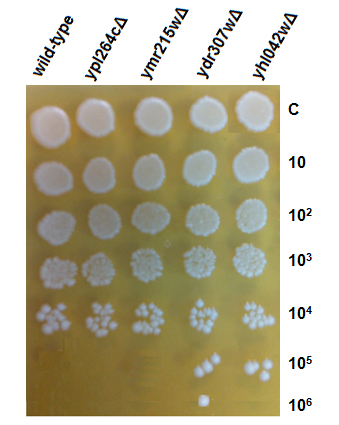

Supplement: Additional file 10: Figure S4. — Colony-forming ability of S. cerevisiae cultures. [file s12918-014-0090-6-S10.tiff]

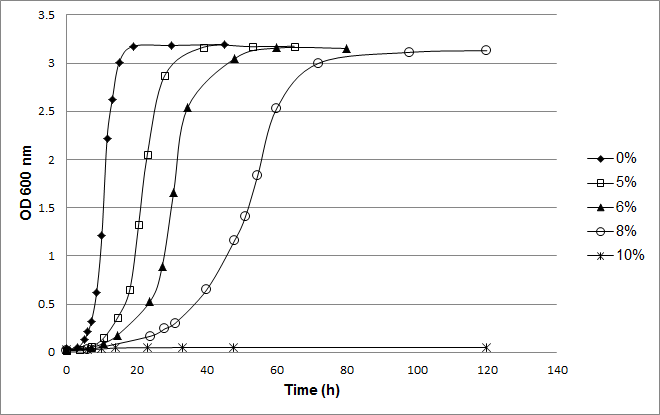

Supplement: Additional file 11: Figure S5. — Growth of the wild type strain in YPD supplemented with different concentrations of ethanol. [file s12918-014-0090-6-S11.tiff]

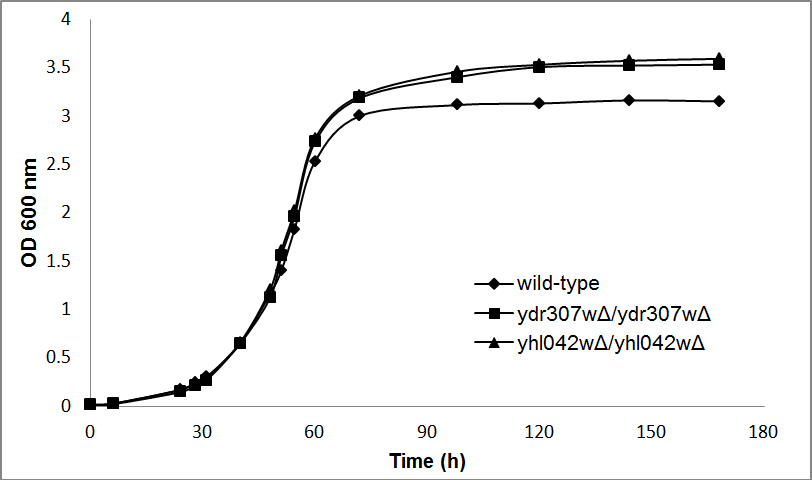

Supplement: Additional file 12: Figure S6. — Growth of S. cerevisiae strains in YPD supplemented with 8% (v/v) ethanol. [file s12918-014-0090-6-S12.tiff]
