# Supplementary material for: A system based network approach to ethanol tolerance in Saccharomyces cerevisiae
Source: BMC Syst Biol. 2014 Aug 8;8:90. doi: 10.1186/s12918-014-0090-6 (PMC4236716; doi:10.1186/s12918-014-0090-6)
Supplement: Additional file 5: Table S4. — Topological properties of the reconstructed networks. [file s12918-014-0090-6-S5.docx]

**Table S4.** Topological properties of the reconstructed networks

| **Model** | **node #** | **edge #** | **D** | **CC** | **CPL** |
| --- | --- | --- | --- | --- | --- |
| ETN (*S. cerevisiae*) | 1933 | 7569 | 10 | 0.166 | 3.7 |
| tETN (*S. cerevisiae*) | 1783 | 7037 | 10 | 0.166 | 3.7 |
| Biogrid (*S. cerevisiae*) | 5517 | 56035 | 6 | 0.245 | 2.7 |

D is diameter, CC is clustering coefficient, CPL is characteristic path length
